# Social engagement and cognitive function among middle-aged and older adults: gender-specific findings from the Korean longitudinal study of aging (2008–2018)

**DOI:** 10.1038/s41598-021-95438-0

**Published:** 2021-08-05

**Authors:** Sarah Soyeon Oh, Eunhee Cho, Bada Kang

**Affiliations:** Mo-Im Kim Nursing Research Institute, Yonsei University College of Nursing, 50-1 Yonsei-ro, Seodaemun-gu, Seoul 03722 Republic of Korea

**Keywords:** Psychology, Diseases, Gastroenterology, Health care, Health occupations, Medical research, Neurology

## Abstract

Recent findings suggest that social disengagement in later life may result in cognitive decline and increase risk of Alzheimer’s and related dementias. However, little is known regarding the gender-specific longitudinal association between social engagement and cognition among middle-aged and older adults. Using data from a nationally representative sample of 2707 men and 5196 women from the Korean longitudinal study of aging, we examined the gender-specific association between social activity and cognitive function. Results from the generalized estimating equation model showed that compared to individuals with consistent social engagement (religious, senior center, sport, reunion, voluntary, political), individuals with inconsistent engagement had lower cognitive function. Transitioning from engagement to non-engagement was associated with lower cognitive function among men only. Not being part of a senior center was associated with decreased cognitive function among both genders, while not being part of a religious group was significant for women only. While marital status was a significant predictor of cognitive ability for women, depression was a significant predictor for men. These findings have implications for policy-makers as interventions targeting improved cognitive function among middle-aged and older adults may be more effective when gender-specific predictors are taken into consideration.

## Introduction

Alzheimer’s disease and related dementias are a group of conditions associated with the decline in an individual’s ability to perform daily activities, primarily in later life^1^. In 2020, it was estimate that more than 50 million people worldwide live with dementia, and that this number will increase three-fold by 2050 with population aging^2,3^. Scholars believe that related societal costs will exceed 1% of global gross domestic product (GDP), equivalent to $818 billion (US dollar) annually^3^. In South Korea, the proportion of older adults with dementia increases by around 27% each year, which is much higher than the global average of 17%^4^. As one of the most rapidly aging societies in the world, this figure is predicted to increase as South Korea becomes a “super-aged society” by 2029, whereby more than 20% of the population consists of older adults^5^.

To date, twelve potentially modifiable risk factors are considered to trigger neuropathological developments across the life course and account for 40% of all dementia cases: (1) less education, (2) hypertension, (3) hearing impairment, (4) smoking, (5) excessive drinking, (6) obesity, (7) depression, (8) physical inactivity, (9) air pollution, (10) traumatic brain injury, (11) diabetes, and (12) low social contact^6,7^.

On the other hand, consistent participation in religious activities, friendship groups, leisure/sports clubs, and/or frequent face-to-face contact with one’s children have been associated with cognitive improvement, even when activities of daily living (ADL), instrumental activities of daily living (IADL), and presence of chronic illnesses are controlled for^8^. Thus, interactions with others have been employed in various interventions to enhance cognitive reserve or resilience, and thereby prevent or delay the incidence of dementia^7^. For example, in a longitudinal study of Japanese community-dwelling older adults, individuals encouraged to engage in daily conversations with friends/relatives had 0.56 times decreased risk of dementia than their counterparts^9^. In the China Health and Retirement Longitudinal Study, those who cared for their grandchildren or participated in voluntary activities also had decreased risk of dementia incidence^10^.

Surprisingly, despite ample evidence regarding the association between social engagement and cognitive function, few studies have focused on gender differences between men and women with cognitive decline^7^. Furthermore, existing studies on this topic have been inconsistent. For example, in an American sample of non-demented, community-dwelling older adults, social support was associated with cognitive decline among men only^11^. Contrastingly, in a Japanese longitudinal study of community-dwelling older adults, social participation was associated with cognitive decline among women only^12^. However, it is challenging to identify key factors that drive the inconsistent findings based on limited number of studies available.

Among Asian populations, researchers have suggested that cognitive ability among older adults differ dramatically by gender, especially among cultures where more educational or economic opportunities were given to boys than girls in the past^13^. Considering South Korea’s deep-rooted history of son preference and patrilineality^14^, we presumed that gender-specific differences would also emerge among middle-aged and older adults in our study. Thus, this study employed the Korean longitudinal study of aging (KLoSA), a large, nationally representative, longitudinal study of community-dwelling Korean adults, to investigate the association between social engagement and cognitive function, according to gender.

## Methods

### Data source and sample

This study employed KLoSA data ranging from 2008 to 2018. The KLoSA is an on-going, biennial panel survey which has been conducted every two years by the Korean Employment Insurance Fund of the Ministry of Labor, following Korea’s transition to an *aging society* in the year 2000^15^. The primary purpose of the study is to examine the demographics, family relationships, health status, health utilization behaviors, employment status, and financial power of middle-aged and older Korean adults over time. The survey sample consists of approximately 10,000 adults over the age of 45, who reside in South Korea (excluding Jeju Island). In 2006, the KLoSA selected 6171 baseline households based on multistage stratified sampling by geographical region (according to the South Korean national census) and household type (apartment/house). For the survey, computer assisted personal interviewing (CAPI) techniques were employed to trace the characteristics of baseline participants over time, which allowed for automatic calculation and scoring of cognition-related questions. Detailed information about the study is available on the KLoSA website (http://survey.keis.or.kr). In our study, we included all individuals over the age of 45 within the KLoSA, who were surveyed from 2008 to 2018. A total of 2707 men and 5196 women over the age of 60 were included in the current analyses. The study was approved by the Institutional Review Board of Yonsei University Hospital (No. 4-2021-0120).

### Study variables

#### Dependent variable: cognitive function

Cognitive function was calculated via the Korean version of Mini Mental State Examination (K-MMSE). The K-MMSE assesses orientation, recall, language, registration, attention, calculation, and the ability to follow simple commands, with a sensitivity of 0.70–0.83 in detecting dementia^16^. The total score ranges from 0 to 30, with higher scores indicating better cognitive function. K-MMSE has been validated in Korean sample with high concurrent validity with another brief measure of cognitive functioning (Bless Orientation Memory Information r = 0.78)^16^.

#### Independent variable: a change in social engagement

In the KLoSA, respondents’ engagement in social activities was assessed by the following question: “Are you participating in the following organization(s)?” Social engagement was categorized by participation in any of the following activities: religious, senior center, leisure, culture, or sport, family or school reunion, volunteer, and political. Using an approach similar to that of a previous study^17^, we employed a linear interpolation data step using the LAG function in SAS, to return adjacent values for social engagement stored previously within our study period. Changes in social engagement were defined as a sudden change in survey response, relative to a consecutive number of unanimous responses in the lag queue. Change in social engagement was classified into the following four categories: (1) consistently engaged, (2) non-engaged to engaged, (3) engaged to non-engaged, and (4) consistently non-engaged. For example, “consistently engaged” indicates the consistent social engagement in 2008 and 2010, in 2010 and 2012, in 2012 and 2014, in 2014 and 2016, and in 2016 and 2019; and “non-engaged to engaged” indicates non-engagement in social activities in 2008 (2010, 2012, 2014, or 2016) than engagement in 2010 (2012, 2014, 2016, or 2018)^17^.

#### Covariates

We included multiple covariates including age, socio-economic status (SES), psychosocial and behavioral factors, and health status in the analyses. SES included income and home ownership. Psychosocial factors included marital status, household size, and having depressive symptoms. We generated a dichotomous variable to assess whether respondents had significant depressive symptoms based on the 10-item short-form Center for Epidemiological Studies-Depression (CES-D-10) with a cutoff score of 10. Behavioral factors included drinking and smoking status, and health-related factors included self-reported health status (from very bad to very good) and number of chronic illnesses (history of clinical diagnosis of hypertension, diabetes, cancer, chronic lung disease, chronic hepatitis, cerebrovascular diseases, mental diseases, and/or arthritis).

### Statistical analysis

Descriptive statistics were used to summarize sample characteristics at baseline as frequencies (%, N). To prevent longitudinal and sampling biases resulting from systemic sampling, weights were provided by KLoSA for each wave, to produce unbiased parameter estimates. For the multiple imputation of missing values, hot decks (original, bounded, generalized estimating equation (GEE), regression) were selected based on a modified predictive mean matching technique using SAS Macro. Effect sizes were estimated using Cohen’s *d* techniques. Analysis of variance (ANOVA) was used to analyze the distribution of K-MMSE scores over the entire time period in association with social engagement. Sociodemographic characteristics of study participants and average K-MMSE scores were calculated for the study period. Our data consisted of multiple imputed values calculated by the makers of the KLoSA research group, to minimize bias, which consisted of 8.5% of missing data on income characteristics. The hot-deck method with modified predictive mean matching was used because of the high proportion of missing data regarding this characteristic. A weighted, GEE model was used to analyze the association between change in social engagement and cognitive function for men and women. All analyses were conducted using SAS version 9.4 (Cary, NC).

### Ethics declaration

All procedures performed in studies involving human participants were in accordance with the ethical standards of the institutional and/or national research committee and with the Declaration of Helsinki (1964) and its later amendments or comparable ethical standards. The study was approved by the Institutional Ethics Board of Yonsei University Hospital (No. 4-2021-0120).

## Results

The detailed breakdown of participants’ baseline characteristics and average K-MMSE scores for each independent variable at baseline by gender are summarized in Table 1. A sample of 2707 Korean men (mean age = 67.86, standard deviation (SD) = 490.41) and 5196 Korean women (mean age = 72.49, standard deviation (SD) = 463.54), from 2008 to 2018, were measured. Among middle-aged and older adults with consistent engagement in social activities, mean K-MMSE scores were 25.05 and 21.33 for men and women respectively, which corresponded to a medium effect size in favor of men (Cohen’s *d* = 0.64, 95% CI 0.46–0.81). Contrastingly, mean K-MMSE scores were 23.30 and 20.78 for men and women respectively among those with consistent non-engagement, which corresponded to a small effect size in favor of men (Cohen’s *d* = 0.34, 95% CI 0.29–0.40).

**Table 1:** Sociodemographic characteristics of study participants and average K-MMSE scores (2008–2018).

	Men				Women			
	n	K-MMSE	SD	*p*-value	n	K-MMSE	SD	*p*-value
**Social engagement**								
Consistently engaged	171	25.05	4.48	< .0001	495	21.33	6.23	< .0001
Non-engaged to engaged	130	24.07	5.68		300	21.32	6.22	
Engaged to non-engaged	129	23.16	7.37		267	21.48	6.73	
Consistently non-engaged	2277	23.30	7.08		4134	20.78	7.44	
**Age group**								
< 60	429	25.82	5.20	< .0001	605	26.17	3.94	< .0001
60–69	991	25.04	5.67		958	24.15	5.14	
70–79	980	22.22	6.93		1869	20.52	6.39	
≥ 80	736	19.32	7.18		1764	15.87	7.30	
**Marital status**								
Married	2167	23.22	7.01	< .0001	2273	22.91	6.35	< .0001
Separated/Divorced	263	24.54	6.29		187	23.70	6.20	
Widowed	277	22.84	6.50		2736	18.76	7.50	
**Income**								
None	1872	21.96	7.42	< .0001	4274	20.16	7.39	< .0001
Low	348	23.93	5.42		587	21.98	5.71	
Medium	233	26.25	4.05		216	25.87	4.69	
High	254	27.25	2.94		119	26.51	3.90	
**Education**								
< Primary	766	23.89	5.75	< .0001	2850	26.50	2.12	< .0001
Secondary	460	26.46	4.27		828	22.11	6.42	
Tertiary	964	27.37	3.68		1244	26.48	3.90	
> Tertiary	517	27.97	3.27		274	27.70	3.14	
**Home ownership**								
Yes	715	24.74	6.61	< .0001	1243	22.22	7.39	< .0001
No	1992	22.81	6.99		3953	20.40	7.18	
**Household size (person/people)**								
1	323	24.34	6.32	< .0001	1316	19.73	6.78	< .0001
2	1421	22.92	6.97		2000	21.59	6.92	
≥ 3	963	23.67	6.99		1880	21.01	7.83	
**Depression**								
Yes	2650	23.54	6.84	< .0001	5099	20.94	7.21	< .0001
No	57	16.92	8.67		97	18.87	8.43	
**Self-reported health**								
Very good	23	27.23	4.86	< .0001	41	23.41	7.96	< .0001
Good	398	26.42	3.67		481	24.76	5.80	
Average	814	25.03	5.00		1654	22.62	6.20	
Bad	1014	22.47	6.58		2218	20.16	6.84	
Very Bad	458	17.58	9.10		802	15.20	8.13	
**Chronic illness**								
0	625	24.90	5.70	< .0001	993	22.65	7.13	< .0001
1	685	24.35	5.86		1326	22.07	6.82	
≥ 2	1397	22.01	7.58		2877	19.63	7.30	
**Drinking status**								
Non-drinker	659	22.04	7.51	< .0001	4116	20.53	7.32	< .0001
Past drinker	1107	24.81	5.19		590	23.46	5.92	
Current drinker	941	22.25	7.88		490	20.11	7.35	
**Smoking status**								
Non-smoker	829	22.46	7.77	< .0001	4770	20.86	7.20	0
Past smoker	1063	22.72	7.07		185	20.42	8.34	
Current smoker	815	24.74	5.44		241	21.98	7.12	
**Year**								
2008	402	24.14	6.19	< .0001	724	21.98	6.68	< .0001
2010	462	23.65	6.89		886	20.85	7.16	
2012	593	24.43	6.67		1102	21.66	7.22	
2014	413	22.98	7.26		855	20.29	7.53	
2016	440	22.96	6.80		863	20.55	7.02	
2018	397	21.73	7.60		766	19.90	7.61	
**Total**	2707	23.41	6.92		5196	20.90	7.24	

Table 2 presents the results of the GEE model which assessed the association between change in social engagement and cognitive function. As shown in the final adjusted GEE models, both men and women who consistently did not engage in social activities (religious, senior center, sport, reunion, voluntary, political) had significantly lower cognitive function than those who consistently engaged in social activities (β = − 1.987, *p* = 0.000 in men; β = − 1.743, *p* < 0.0001 in women). While marital status had no association with cognition in men, widowed women (β = − 0.699, *p* = 0.002) had lower cognitive function than married women, as did women living in households of three or more people (β = − 0.524, *p* = 0.011). Individuals who had depressive symptoms demonstrated poorer cognitive function than those not having depressive symptoms among men only (β = − 2.994, *p* = 0.001). Additionally, while both men and women who reported poor self-rated health exhibited significantly poorer cognitive function than those who self-rated health status as very good (β = − 6.583, *p* < 0.001 in men; β = − 4.71, *p* < 0.001).

**Table 2:** GEE analysis of association between social engagement and cognitive decline measured via K-MMSE.

	Men			Women		
	β*	S.E	*p*-value	β*	S.E	*p*-value
**Social engagement**						
Consistently engaged	Ref			Ref		
Non-engaged to engaged	− 0.656	0.788	0.405	− 0.496	0.446	0.266
Engaged to non-engaged	− 1.570	0.804	0.051	− 0.686	0.470	0.144
Consistently non-engaged	− 1.987	0.555	0.000	− 1.743	0.302	< .0001
**Age group**						
< 60	Ref			Ref		
60–69	− 0.283	0.345	0.412	− 1.268	0.297	< .0001
70–79	− 2.155	0.386	< .0001	− 4.216	0.307	< .0001
≥ 80	− 4.414	0.456	< .0001	− 8.302	0.340	< .0001
**Marital status**						
Married	Ref			Ref		
Separated/Divorced	− 0.341	0.392	0.385	0.007	0.422	0.986
Widowed	− 0.027	0.459	0.954	− 0.699	0.226	0.002
**Income**						
None	− 2.003	0.415	< .0001	− 1.125	0.489	0.021
Low	− 1.158	0.490	0.018	− 1.146	0.533	0.032
Medium	− 0.387	0.480	0.420	0.158	0.582	0.787
High	Ref			Ref		
**Education**						
< Primary	− 1.996	0.31	< .0001	− 2.53	0.329	< .0001
Secondary	0.371	0.341	0.2763	− 0.886	0.376	0.0186
Tertiary	Ref			Ref		
> Tertiary	0.18	0.432	0.6766	0.044	0.825	0.9568
**Home ownership**						
Yes	Ref			Ref		
No	1.172	0.264	< .0001	1.057	0.199	< .0001
**Household size (person/people)**						
1	0.666	0.407	0.102	− 0.146	0.250	0.560
2	Ref			Ref		
≥ 3	− 0.140	0.261	0.591	− 0.524	0.206	0.011
Depression*						
Yes	− 2.994	0.886	0.001	− 0.201	0.674	0.765
No	Ref			Ref		
**Self-reported health**						
Very good	Ref			Ref		
Good	− 0.306	1.136	0.788	1.491	0.949	0.116
Average	− 0.832	1.126	0.460	0.763	0.928	0.411
Bad	− 2.632	1.136	0.021	− 0.641	0.930	0.490
Very Bad	− 6.583	1.181	< .0001	− 4.741	0.956	< .0001
**Chronic illness**						
0	0.617	0.327	0.059	1.142	0.259	< .0001
1	0.316	0.321	0.325	0.645	0.246	0.009
≥ 2	Ref			Ref		
**Drinking status**						
Non-drinker	Ref			Ref		
Past drinker	0.799	0.327	0.015	0.391	0.260	0.133
Current drinker	0.640	0.344	0.063	− 0.140	0.298	0.637
**Smoking status**						
Non-smoker	Ref			Ref		
Past smoker	0.366	0.319	0.252	0.957	0.477	0.045
Current smoker	0.541	0.340	0.111	0.797	0.397	0.045

*Sum of CESD-10 ≥ 10 (Lee et al. 2014).

Table 3 presents the results of the subgroup analysis which discerned which type of social activities are most associated with cognitive function. Regarding type of social activities, consistent non-engagement in a senior center was associated with significant poorer cognitive function among both men (β = − 1.987, *p* = 0.000) and women (β = − 1.743, *p* < 0.0001) compared to those who consistently engaged in a senior center. In women only, individuals in the group of consistent non-engagement in religious activities exhibited lower cognitive function with the reference group of consistent engagement in religious activities (β = − 2.039, *p* = 0.027). Social engagement in sports groups was not associated with cognitive function in our study population, and information on political, volunteer, and reunion-related activities were too small in number for our subgroup analyses when stratified by gender.

**Table 3:** Subgroup analysis of association between type of social engagement and cognitive decline measured via K-MMSE.

	Men			Women		
	β*	S.E	*p*-value	β*	S.E	*p*-value
**Type of social engagement**						
Religious						
Consistently engaged	Ref			Ref		
Non-engaged to engaged	0.946	1.910	0.620	0.383		
Engaged to non-engaged	− 0.786	1.899	0.679	− 1.398	1.154	0.226
Consistently non-engaged	− 2.556	1.451	0.078	− 2.039	0.924	0.027
Senior Center						
Consistently engaged	Ref			Ref		
Non-engaged to engaged	− 0.656	0.788	0.405	− 0.496	0.446	0.266
Engaged to non-engaged	− 1.570	0.804	0.051	− 0.686	0.470	0.144
Consistently non-engaged	− 1.987	0.555	0.000	− 1.743	0.302	< .0001
Sport						
Consistently engaged	Ref			Ref		
Non-engaged to engaged	− 0.353	5.652	0.950	0.101	2.574	0.969
Engaged to non-engaged	1.287	5.679	0.821	0.759	2.623	0.772
Consistently non-engaged	− 1.942	5.388	0.719	− 1.378	2.315	0.552
Reunion						
Consistently engaged	–			–		
Non-engaged to engaged	–			–		
Engaged to non-engaged	–			–		
Consistently non-engaged	–			–		
Volunteer						
Consistently engaged	–			–		
Non-engaged to engaged	–			–		
Engaged to non-engaged	–			–		
Consistently non-engaged	–			–		
Political						
Consistently engaged	–			–		
Non-engaged to engaged	–			–		
Engaged to non-engaged	–			–		
Consistently non-engaged	–			–		

## Discussion

Given that previous studies examined social engagement at a single point in time and did not stratify subjects by gender, our study provides unique, longitudinal information on the gender-specific association between changes in social engagement over time and cognitive function among middle-aged and older Korean adults. Our results support existing evidence that social engagement is an important protective factor for preventing cognitive decline or dementia in later life^17,18^. While the normal range of the K-MMSE is recognized as ≥ 24, our study population’s K-MMSE scores (male: 25.05, female: 21.33) were lower than that of the general South Korean population. However, our figures were in alignment with that of previous studies which have used the same KLoSA dataset to measure cognitive function via the K-MMSE among Korean males and females (male: 24.8; female: 21.2), implying that the individuals sampled in the dataset may have differing K-MMSE scores on average than that of the general population^19^.

In our study, the magnitude of the association between social disengagement and poor cognitive function was stronger among men than women. While both genders were significantly affected by consistent social disengagement, transitioning from engagement to non-engagement was a predictor of cognitive decline among men only. Such findings support the claims of previous studies that have found that risk of social isolation increases four-fold in older men than women^20^.

Similarities and differences were found beween men and women regarding the association between change in social engagment over time and cognitive function. Consistent social engagement via membership in a senior center was protective of cognitive decline for both men and women. This finding is consistent with a prior study of Korean middle-aged and older adults, in which participation in senior center programs (e.g., South Korean Dementia Prevention Program on Cognitive and Emotional Functions) was associated with improved cognitive/physical function and emotional wellbeing among both genders^21^.

Globally, senior centers, also known as ‘elderly centers’ or ‘seniors’ social clubs’, have played a key role in increasing social interaction and promoting active aging among community-dwelling middle-aged and older adults^22–25^. In senior centers, a wide variety of free services for physical and mental health improvement, as well as social/educational programs and recreational activities are organized by healthcare professionals, social workers, and community volunteers^25,26^. In Korea, senior centers offer opportunities for middle-aged and older adults to socially interact with other people while playing chess or ‘*Hwatu’* (Korean traditional cards), singing, dancing, playing musical instruments, and learning calligraphy or origami^27,28^.

As explained by Lee and Kim^27^, the association between senior center participation and improved cognitive function may be attributed to the enhancement of various cognitive reserves^29^. That is, cognitive and emotional stimulations via intellectually challenging activities and interpersonal exchanges with others may enhance certain cognitive reserves in the brain, which allow individuals to better cope with age-related brain change or pathological changes before cognitive impairment is manifested^27,29^. However, as emphasized by psychologists and brain scientists in previous studies, more information including neuroimaging data in clinical studies with long-term follow-up, is necessary to understand the exact, underlying mechanisms behind successful cognitive decline over time^30^.

Consistent engagement in religious activities was also associated with higher cognitive function than consistent non-engagement for women only. While the exact mechanisms behind this phenomenon are unknown, religious activities (e.g. listening to sermons, reciting prayers, singing hymns) may stimulate various domains of intelligence (e.g. verbal, musical, emotional etc.) and inhibit cognitive decline^31^. However, more studies are needed to determine the underlying mechanisms whereby cognitive health can accrue from the different types of social/physical/mental activities in middle-aged and older adults.

Likewise, in our study, we found that engagement in social activities precedes cognitive decline; which resulted in our assumption that social engagement is a causal factor rather than consequence of Alzheimer changes. However, many scholars claim that long before dementia is diagnosed, there is a progressive reduction in various mental and physical activities, as depositions of amyloid and tau accumulate in the brain over time^32,33^. For example, in the UK Million Women Study which had a mean follow-up of 16 years, Floud and colleagues found that physical inactivity was only associated with dementia rates during the first few years of follow-up, and not associated with rates in the second decade^32^. Such results provide evidence that being active has little or no protective effects against clinically apparent dementia, and may be a consequence rather than cause of decreased cognitive function. More prospective studies with long-term follow-up are needed to examine the reliability of this claim^32^.

Several limitations of this study should be noted. For longitudinal studies that target older adults, the loss of study participants over time will inevitably result in substantial bias, especially in studies that investigate health-related outcomes^34^. In the case of the Korea Longitudinal Study of Aging dataset, previous publications of cognitive trajectories have shown a declination rate every 2 years of around − 1.6 to − 1.2 in the K-MMSE over a period of 6 years^35^. In our dataset, there were similar trends over the course of 10 years; however, further studies exceeding a decade are necessary for an accurate understanding of longitudinal and causational trends. Also, while multiple imputed values based on the hot-deck method with modified predictive mean matching were used to control for missing data, it is likely that individuals with substantial cognitive decline were lost during the follow-up period due to illness and/or death.

Although the Korea Longitudinal Study of Aging employed sampling methods representative of national age and gender distributions, as well as geographic localizations of South Korea’s 16 major metropolitan cities and provinces, our study is likely to have resulted in a disproportionately higher number of subjects with certain characteristics (i.e. female, higher socioeconomic status etc.) than national proportions because of survival bias. To overcome such limitations, the survey makers did provide weights reflecting population distribution changes over time, as well as non-response-adjusted weights, and benchmark weights by year for researchers of longitudinal analyses^36^. However, readers should take into account that our study sample may not be a proportionately accurate representation of South Korea’s aging population.

Social engagement was measured via participation in any of the social organizations, which were grouped into six categories (i.e., religious, senior center, sport, reunion, volunteer, and political). Not only were we unable to control for the frequency of such engagements, but it was presumed that participation in all of these organizations would be of equal influence to the cognitive function of individuals. It is recommended that in future studies, such actions are given weights, especially when taking a gender-specific approach because certain actions may have more influence over others depending on various sociodemographic characteristics.

Lastly, we were unable to control for a range of factors that may have affected the social interaction of these individuals because they were not measured in the initial survey questionnaire. Social interactions among older adults may be affected by life circumstances, the presence or absence of children/grandchildren, an individual’s degree of attachment to their local community setting^37^, and increasingly, an individual’s level of digital literacy and online social networks^38^. Thus, additional studies are needed to examine further the factors that may be contributing to the gender differences in the association between social engagement and cognitive function among community-dwelling middle-aged and older adults.

Despite such limitations, our study is unique because it longitudinally assessed the gender-specific effect of change in social engagement over time on cognitive function among older adults. To our knowledge, our study is one of the first studies of its kind in South Korea, and relays the importance of consistent engagement in social activities across later life in both men and women to prevent and delay cognitive decline. These findings have implications that are crucial to community interventions and policies that promote cognitive function in older adults. Future interventions should develop tailored social engagement programs by considering their gender, sociodemographic characteristics, health status, health-related behaviors, and social interests or preferences in specific types of activities (religious, senior center, sport etc.). The distinct results between men and women on the association between social engagement and cognition should be confirmed by future studies that account for other potential confounders such as social network size and density, stress level, stressful life events, and perceived social support or loneliness.

## Data Availability

The datasets generated during and/or analysed during the current study are available in the [KLOSA] repository, [https://survey.keis.or.kr/klosa/klosa01.jsp].
